# Frequency and predictors of estimated HIV transmissions and bacterial STI acquisition among HIV-positive patients in HIV care across three continents

**DOI:** 10.7448/IAS.19.1.21096

**Published:** 2016-09-28

**Authors:** Steven A Safren, James P Hughes, Matthew J Mimiaga, Ayana T Moore, Ruth Khalili Friedman, Kriengkrai Srithanaviboonchai, Mohammed Limbada, Brian D Williamson, Vanessa Elharrar, Vanessa Cummings, Jessica F Magidson, Charlotte A Gaydos, David D Celentano, Kenneth H Mayer, for the HPTN063 Study Team

**Affiliations:** 1 The Fenway Institute, Fenway Health, Boston, MA, USA; 2 Department of Psychology, University of Miami, Coral Gables, FL, USA; 3 Fred Hutchinson Cancer Research Center, Seattle, WA, USA; 4 School of Public Health, University of Washington, Seattle, WA, USA; 5 Institute for Community Health Promotion, Brown University, Providence RI, USA; 6 FHI360, Durham, NC, USA; 7 Instituto Nacional de Infectologia Evandro Chagas, FIOCRUZ, Rio de Janeiro, Brazil; 8 Research Institute for Health Sciences, Chiang Mai University, Chiang Mai, Thailand; 9 Faculty of Medicine, Chiang Mai University, Chiang Mai, Thailand; 10 Centre for Infectious Disease Research in Zambia, Lusaka, Zambia; 11 National Institute of Allergy and Infectious Disease (NIAID), Bethesda, MD, USA; 12 Department of Pathology, Division of Infectious Diseases, Johns Hopkins School of Medicine, Baltimore, MD, USA; 13 Department of Medicine, Division of Infectious Diseases, Johns Hopkins School of Medicine, Baltimore, MD, USA; 14 Behavioral Medicine Service, Massachusetts General Hospital, Harvard Medical School, Boston, MA, USA; 15 Department of Epidemiology, Johns Hopkins Bloomberg School of Public Health, Baltimore, MD, USA; 16 Department of Medicine, Harvard Medical School, Beth Israel Deaconess Medical Center, Boston, MA, USA

**Keywords:** treatment as prevention, secondary prevention, MSM, global HIV prevention, global HIV transmission

## Abstract

**Introduction:**

Successful global treatment as prevention (TasP) requires identifying HIV-positive individuals at high risk for transmitting HIV, and having impact via potential infections averted. This study estimated the frequency and predictors of numbers of HIV transmissions and bacterial sexually transmitted infection (STI) acquisition among sexually active HIV-positive individuals in care from three representative global settings.

**Methods:**

HIV-positive individuals (*n=*749), including heterosexual men, heterosexual women and men who have sex with men (MSM) in HIV care, were recruited from Chiang Mai (Thailand), Rio De Janeiro (Brazil) and Lusaka (Zambia). Participants were assessed on HIV and STI sexual transmission risk variables, psychosocial characteristics and bacterial STIs at enrolment and quarterly for 12 months (covering 15 months). Estimated numbers of HIV transmissions per person were calculated using reported numbers of partners and sex acts together with estimates of HIV transmissibility, accounting for ART treatment and condom use.

**Results:**

An estimated 3.81 (standard error, (SE)=0.63) HIV transmissions occurred for every 100 participants over the 15 months, which decreased over time. The highest rate was 19.50 (SE=1.68) for every 100 MSM in Brazil. In a multivariable model, country×risk group interactions emerged: in Brazil, MSM had 2.85 (95% CI=1.45, 4.25, *p*<0.0001) more estimated transmissions than heterosexual men and 3.37 (95% CI=2.01, 4.74, *p*<0.0001) more than heterosexual women over the 15 months. For MSM and heterosexual women, the combined 12-month STI incidence rate for the sample was 22.4% (95% CI=18.1%, 27.3%; incidence deemed negligible in heterosexual men). In the multivariable model, MSM had 12.3 times greater odds (95% CI=4.44, 33.98) of acquiring an STI than women, but this was not significant in Brazil. Higher alcohol use on the Alcohol Use Disorders Identification Test (OR=1.04, 95% CI=1.01, 1.08) was also significantly associated with increased STI incidence. In bivariate models for both HIV transmissions and STI incidence, higher depressive symptoms were significant predictors.

**Conclusions:**

These data help to estimate the potential number of HIV infections transmitted and bacterial STIs acquired over time in patients established in care, a group typically considered at lower transmission risk, and found substantial numbers of estimated HIV transmissions. These findings provide an approach for evaluating the impact (in phase 2 studies) and potentially cost-effectiveness of global TasP efforts.

## Introduction

Treatment as prevention (TasP) is part of a major strategy to curtail the HIV epidemic globally, especially in the light of recent studies finding that the odds of sexual transmission of HIV to one’s uninfected partner can be reduced or potentially eliminated by using antiretroviral therapy (ART) to suppress the viral load of the infected partner [1,2]. HIV care is a setting where people living with HIV/AIDS (PLWHA) who could potentially transmit HIV can be identified and offered secondary HIV prevention interventions as a part of TasP. To evaluate secondary prevention interventions, it is important to be able to estimate the potential impact on estimated number of infections that different risk groups may be transmitting, even in the context of widespread HIV care.

In most HIV secondary prevention studies for PLWHA, the number of self-reported condomless sexual acts is the primary outcome measure [3,4]. However, HIV transmission risk depends on a number of factors, including the number of sexual acts with HIV-negative partners, whether or not condoms are used, whether or not the HIV-positive partner is taking ART and is virally suppressed, and, in the case of anal sex, whether or not the infected individual is the insertive or receptive partner [1,2,5,6]. By combining these factors, it is possible to estimate HIV transmission risk. Using the estimated number of HIV transmissions as a main outcome measure has greater practical and public-health implications for rolling out a TasP programme than using only self-reported condomless sex acts. Frequently used psychosocial predictors of condomless sex (e.g. social support, substance use and depression) and culturally derived potentially relevant factors might be predictive of estimated infections as an outcome.

To inform TasP programmes and ways to evaluate their potential impact, this study sought to examine potential HIV transmissions and the frequency of bacterial sexually transmitted infection (STI) acquisition among a diverse, identifiable group of HIV-positive individuals, including heterosexual men, heterosexual women and men who have sex with men (MSM) in HIV care in three global settings. Additionally, we examined whether relevant psychosocial factors were associated with the estimated number of HIV transmissions and incident bacterial STI in this cohort.

## Methods

### Participants

**Figure 1:**
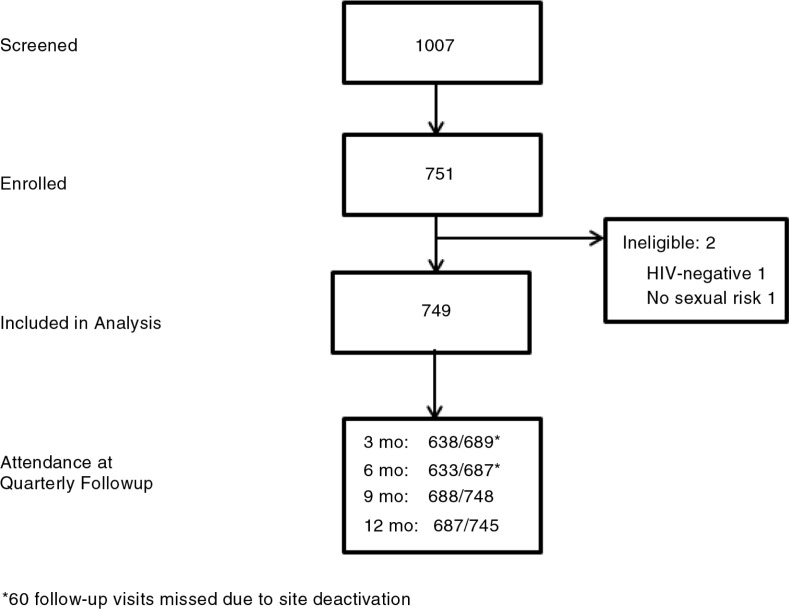
Strobe diagram depicting participant flow.

HPTN063 was a one-year multi-site longitudinal, observational cohort study of high-risk HIV-positive individuals (*n*=749) in HIV care in Thailand (Chiang Mai), Brazil (Rio De Janeiro) and Zambia (Lusaka). All three sites enrolled heterosexual men and women, and MSM were enrolled in Thailand and Brazil (see Figure 1 for enrolment and study flow).

Adults (over 18 years old) were included if they had 1) documented evidence of HIV infection; 2) been receiving HIV care (defined as at least two visits within nine months prior to enrolment at a formal health care setting) and 3) a history of sexual risk behaviour in the past 12 months (defined as either having had an STI, having had vaginal or anal intercourse without a condom, reported difficulty negotiating condom use or non-disclosure of HIV status to an HIV-negative partner or partner of unknown HIV serostatus). (Inclusion criteria were modified with Version 2.0 of the protocol on 31 October 2011 to allow for an expanded definition of “sexual risk behaviour” based on the current literature and anecdotal evidence from the patient population. The original criteria stated: Reported history of unprotected (i.e. without a condom) insertive penile or receptive vaginal or anal intercourse with a sexual partner who is known to be HIV-negative or of unknown HIV status at least once within three months of enrolment.) Participants were excluded if they 1) were enrolled in any other study that included behavioural HIV risk reduction counselling or HIV prevention; 2) reported having unprotected sex for the purpose of conceiving; 3) were planning to relocate from the area in the following year or 4) had any condition that may make participation in the study unsafe or inappropriate as deemed by the primary investigator(s).

Participants in Brazil were recruited from the Instituto Nacional de Infectologia Evandro Chagas Clinical Trials Unit in Rio de Janeiro. In Zambia, participants were recruited from the Matero Reference Clinic Clinical Research Site and George Health Clinic Clinical Research Site in Lusaka, Zambia. In Thailand, participants were recruited from several local HIV clinics and non-governmental organizations by the Research Institute for Health Sciences, Chiang Mai University, though only individuals established in HIV care, as with the other sites, were enrolled.

At all sites, screening and enrolment occurred on the same day or within the following 30 days, depending on availability of participants and clinic staff. If a participant provided informed consent and met eligibility criteria for the study, then he/she was enrolled. In Brazil, enrolment opened on 30 March 2011 and closed on 6 April 2012, with the last follow-up occurring on 27 May 2013. In Thailand, enrolment opened on 26 March 2010, was paused due to technical challenges with the data capture device on 22 June 2010, resumed enrolment on 8 February 2011 and completed enrolment on 19 April 2012, with study closure on 2 May 2013. In Zambia, enrolment opened on 18 April 2011 and closed on 19 April 2012 with the last follow-up visit occurring on 17 April 2013.

All procedures were approved by the local and relevant institutional review boards. For Zambia, this included the IRB for the University of Zambia Biomedical Research Ethics Committee and the University of Alabama at Birmingham IRB. For Brazil, this included the Comite de Etica em Pesquisa do Instituto de Pesquisa Clinica Evandro Chagas and the National Commission of Ethics in Research (CONEP). For Thailand, this included the Human Experimentation Committee, Research Institute for Health Sciences, Chiang Mai University and the Committee on Human Research, Johns Hopkins Bloomberg School of Public Health.

### Procedures

There were five assessments over the course of one year: baseline, and then quarterly, to generally correspond to timing of HIV care visits. Items were translated, back translated by someone who did not have access to the original English forms and checked for accuracy by study staff and the first author. Languages included Portuguese (Brazil), Thai (Thailand), Bemba and Nyanja (Zambia). In addition to basic demographic information, participants completed the following assessments, on the same visit, with an interviewer, except for the sexual behaviour assessment that was done via audio computerized self-assessment (ACASI) as described below.

*Substance use* was assessed via interview using a range of locally used substances at each site (e.g. marijuana, opioids, cocaine, steroids, methamphetamine, sildenafil (Viagra) and female hormones). Responses were dichotomized as affirmative for use of any drug in the past three months.

*Alcohol use* was measured via interview using the Alcohol Use Disorders Identification Test [7], with a standard cut-off of eight reflecting hazardous and harmful alcohol use and possible alcohol dependence.

*ART adherence* was assessed using an interviewer-administered questionnaire [8] that assessed “how would you rate your adherence” according to six ordinal categories for ratings adherence (very poor, poor, fair, good, very good and excellent).

*Social support* was measured via interview using nine items from the Multidimensional Scale of Perceived Social Support (MSPSS) [9], which assessed social support across family, friends and partners. Higher scores reflect greater social support.

*Depressive symptoms* were measured via interview using the Center for Epidemiologic Studies Depression Scale [10]. A score of ≤7 is considered as no depressive symptoms, 8 to 15 as mild to moderate depressive symptoms and ≥16 as severe depressive symptoms.

*Quality of life* was assessed using the interview administered ACTG SF-21 [11,12], which has strong psychometric properties for assessing quality of life for PLWHA. There are eight subscales: physical functioning, role functioning, pain, current health perceptions, emotional well-being, cognitive functioning, energy/fatigue and social functioning. Scores were standardized, with a possible range of 1 to 100 with higher scores reflecting better functioning.

#### HIV disclosure

With input from each site and their community advisors, HIV disclosure was assessed by interviewers using six items developed for this study assessing how the participant felt about disclosing their HIV status. Responses were assessed on a 4-point Likert scale ranging from disagree strongly to agree strongly. Principal component 1 involved disclosure behaviours and principal component 2 centred around fear of consequences of disclosure. Higher scores indicate less disclosure of HIV status and greater fear related to consequences of disclosure, respectively [13].

#### Cultural factors: perceived community beliefs and personal belief

Similar to the disclosure scale, with input from each site and their community advisors, we generated a list of 23 potential perceived community beliefs and 16 potential perceived personal beliefs that might be associated with HIV transmission behaviour. We conducted a principal components analysis on each of the two scales, which yielded four components each (see Supplementary file). For community beliefs, the four principal components centred around 1) power imbalance and condom negotiation, 2) sexual expectations and beliefs, 3) negative community beliefs about HIV and 4) sexual immorality. For personal beliefs, the four principal components generally centred around 1) sexual practices, 2) partner concerns, 3) viral load beliefs and 4) dislike of condoms. Higher values indicate stronger agreement with the factor.

#### Condomless sex

Condomless sex was assessed via ACASI to minimize social desirability bias [14,15]. Insertive or receptive (vaginal and/or anal) intercourse with up to five partners in the past three months was assessed. For each, participants were asked about HIV status (HIV-negative, HIV-positive or HIV-not known) and whether they had vaginal or anal sex with that partner. If anal sex, participants were asked if it was insertive or receptive and whether a condom was used. Before addressing technical issues with the ACASI, the first 61 participants (Thailand only) were asked about all partners in the past three months, however, but only five reported more than five partners.

#### Bacterial sexually transmitted infections

Gonorrhoea and chlamydia were assessed via nucleic acid amplification tests (NAATs) (GenProbe/Hologic, SanDiego, CA) using urine for heterosexual men and self- or clinician-collected rectal swabs and urine for MSM and vaginal swabs (self- or clinician-collected) for women. Collected specimens were stored at each site and later shipped to the HPTN Laboratory Centre at Johns Hopkins University (JHU) in Baltimore, MD, with a subset being tested before the entire sample. If an STI was identified, participants were called back into the clinic for re-testing and treatment. Syphilis serological testing was performed at the local laboratories in real time using blood samples and a screening assay (either rapid plasma reagin (RPR) or venereal disease research laboratory (VDRL)) and confirmed at the local site by a confirmatory assay (using MHA-TP or FTA-ABS), with quality assurance reviewed by the HPTN Laboratory Centre at JHU. Incident syphilis results were reviewed by a panel of expert clinicians to adjudicate endpoints following CDC recommendations [16]. Our STI analysis pools any incident report of gonorrhoea, chlamydia and syphilis.

During a review of STI incidence in the subset initially tested, MSM and heterosexual women had sufficient STI incidence to power the planned analyses. The laboratory, however, found only a 0.3% incidence for the heterosexual men and determined that, due to limited resources, additional samples from heterosexual men would not be analyzed (assuming low incidence).

### Statistical analysis

We summarized continuous measures with median and interquartile ranges. Categorical measures were tabulated. Incidence was computed as total number of events divided by total time at risk.

For participant *i* at visit *t*, we computed the expected number of HIV transmissions over the past three months, *E*_*it*_, as

$$E_{it}=\sum_{k}\sum_{j=1}^{m_{ikt}}1-{\left(1-p_{ik}\right)}^{n_{ijkt}}$$

where *k* indexes the type of act (i.e. unprotected vaginal sex), *m*_*ikt*_ is the number of HIV-negative and/or HIV unknown status partners with whom participant *i* had one or more acts of type *k* over the three months prior to visit *t* (collected from the participant via ACASI), *n*_*ijkt*_ is the number of acts of type *k* participant *i* had with partner *j* over the three months prior to visit *t* (from ACASI) and *p*_*ik*_ is the per act probability of HIV transmission [5,17] for participant *i* and act type *k*. The *p*_*ik*_ depended on the risk group, type of act, whether or not a condom was used and the ART status of the participant (Table 1). In some cases, the reported numbers of acts over the past three months appeared to be incorrectly entered or were unbelievably high. To limit the effect of severe outliers (i.e. 9999 acts in three months), we truncated the number of acts at 212 (the 99th percentile). This corresponds to about 2 1/3 acts per day for a 90-day period. Note that an individual with only HIV-positive partners would have 0 expected transmissions.

**Table 1:** Probability of transmitting HIV in a single act used in modelling expected transmission, by type of sex, ART status of infected participant and condom use

	No ART		ART
		
	Unprotected	Protected	All
MSM insertive	0.014	0.0028	0.0005
MSM receptive	0.007	0.0014	0.0005
Heterosexual	0.001	0.0002	0.00005

MSM=men who have sex with men; ART=antiretroviral therapy.

The *E*_*i*_ is multiplied by 100 to represent the expected number of transmissions per 100 participants per three months. To compute the expected number of transmissions over the entire follow-up period and avoid bias due to loss to follow-up, we fit a linear model with the *E*_*it*_ as the outcome, covariates for quarter, risk group and site (and group×site interaction) and random person effects. By taking linear combinations of model parameters, we computed the mean expected number of transmissions (and standard errors) over the one year follow-up for each risk group–country combination. We also extended the model to include baseline data, which allowed us to compute mean expected number of transmissions over the entire 15-month study period.

Separately, we used the *E*_*it*_ as the outcome in a linear regression analysis to relate the expected number of transmissions to various covariates (univariate analysis) using generalized estimating equations (GEE) linear regression with independence working correlation and clustered by participant. We then selected factors that were significant at the 0.10 level for a multivariable predictive model. Backwards elimination (with risk group and site forced into the model) was used to fit the most parsimonious model.

A similar approach was taken to fit bivariate models and a multivariable model for incident bacterial STI (defined as any incident report of gonorrhoea, chlamydia and syphilis) using GEE logistic regression.

## Results

Baseline descriptive data for the sample were reported in our baseline paper [13] with supplemental information in the Supplementary file. Across risk groups, the median age ranged from low 30s to 40s, with racial dispersions representative of the respective countries of enrolment. Less than half of the samples were never married, with almost all of the MSM reporting never being married, and a range of 22 to 76% of heterosexual women and heterosexual men reporting being married versus not depending on site. Most participants reported at least some schooling, at least at the secondary level.

### Estimated number of HIV transmissions during the study period

Summarizing across the entire 15-month period, there were 3.81 estimated HIV transmissions for every 100 people in the study, although this number is influenced by the very high number of estimated HIV transmissions (19.50) among MSM in Brazil (Table 2). Intermediate numbers of estimated HIV transmissions emerged in MSM in Thailand (3.52), heterosexual women in Brazil (3.20) and heterosexual men in Brazil (2.08). Lower numbers (less than 1) were from heterosexual men in Thailand (0.27) and heterosexual men in Zambia (0.36), heterosexual women in Thailand (0.64) and heterosexual women in Zambia (0.91).

**Table 2:** Estimated HIV transmission risk and STI incidence in the study over time, by risk group and site

	Thailand			Brazil			Zambia		Total
				
	Heterosexual men (*n=*100)	MSM (*n=*100)	Heterosexual women (*n=*100)	Heterosexual men (*n=*64)	MSM (*n=*100)	Heterosexual women (*n=*99)	Heterosexual men (*n=*86)	Heterosexual women (*n=*100)	(*N=*749)
**Estimated HIV transmissions per 100 HIV-positive individuals (SE)**									
Baseline (for the past three months)	0.09 (0.69)	1.02 (0.65)	0.09 (0.76)	0.68 (0.75)	9.33 (0.60)	0.96 (0.60)	0.12 (0.67)	0.22 (0.67)	1.56 (0.24)
For the 12 months after baseline	0.18 (0.87)	2.49 (0.85)	0.56 (0.93)	1.45 (1.11)	9.92 (0.87)	2.21 (0.86)	0.25 (0.90)	0.72 (0.85)	2.22 (0.32)
Over the 15 months assessed	0.27 (1.72)	3.52 (1.67)	0.64 (1.86)	2.08 (2.13)	19.5 (1.68)	3.20 (1.66)	0.36 (1.77)	0.91 (1.69)	3.81 (0.63)
**STI incidence (over 12 month follow-up) (95% CI) [person-years]**		50.4% (36.3%, 68.1%) [83.4]	5.1% (1.7%, 11.9%) [98.1]		34.1% (22.3%, 50.0%) [76.2]	15.2% (7.9%, 26.6%) [78.9]		11.9% (5.9%, 21.2%) [92.7]	22.4% (18.1%, 27.3%) [429.2]

STI=sexually transmitted infection; SE=standard error.

### Predictors of estimated number of HIV transmissions during the 15-month study period

#### Bivariate predictors

The number of estimated transmissions per three months interval decreased by 0.10 per quarter during the study (Table 3). Additionally, over the study period, those who had poorer general health perceptions, worse pain, worse mental health and low energy/fatigue on the ACTG QOL scale had higher numbers of estimated transmissions. Those with higher levels of fear of HIV disclosure (the second principal component on the disclosure scale) and those who endorsed various beliefs on the cultural questionnaire (lower community beliefs about power imbalance/condom negotiation, higher community beliefs about sexual expectations and lower personal beliefs about sexual practices) had higher numbers of estimated transmissions. Those with greater levels of depressive symptoms had higher numbers of estimated HIV transmissions, and those who did not use substances had lower numbers of estimated infections. There were bivariate effects for site, with Brazil having more estimated transmissions than Thailand and Zambia having fewer estimated infections than Thailand. Finally, there were effects for risk group, with MSM having more estimated transmissions than heterosexual men.

**Table 3:** Estimated HIV transmission risk as outcome

HIV transmission risk		Univariate models	
	
Characteristics	Level	Estimate (95% CI)	*p*
**Time (quarter)**		**−0.10 (−0.16, −0.04)**	**0.0012**
**General health**		**−0.19 (−0.37, −0.01)**	**0.0387**
Physical functioning		−0.19 (−0.41, 0.04)	0.1086
Role functioning		−0.23 (−0.49, 0.04)	0.0964
Social functioning		−0.05 (−0.15, 0.05)	0.3027
Cognitive functioning		−0.13 (−0.35, 0.09)	0.2504
**Pain**		**−0.20 (−0.39, 0.00)**	**0.0548**
**Mental health**		**0.21 (−0.40, −0.01)**	**0.0370**
**Energy/Fatigue**		**−0.26 (−0.48, −0.05)**	**0.0167**
Social support		0.04 (−0.02, 0.09)	0.1674
HIV disclosure behaviours		−0.34 (−1.1, 0.41)	0.3703
**Fear of the consequences of HIV disclosure**		**0.66 (0.12, 1.20)**	**0.0164**
Alcohol score		0.03 (−0.01, 0.06)	0.1170
**Community beliefs: power imbalance and condom negotiation**		**−0.27 (−0.49, −0.05)**	**0.0163**
**Community beliefs: relationship dynamics**		**0.42 (0.19, 0.65)**	**0.0004**
Community Beliefs: Community negative beliefs about HIV		−0.14 (−0.56, 0.27)	0.5014
Community Beliefs: Sexual immorality		−0.20 (−0.40, 0.01)	0.0626
**Personal beliefs: sexual practices**		**−0.44 (−0.69, −0.18)**	**0.0008**
Personal beliefs: partner concerns		0.01 (−0.18, 0.20)	0.9403
Personal beliefs: viral load beliefs		−0.12 (−0.54, 0.31)	0.5861
Personal beliefs: dislike of condoms		0.40 (−0.03, 0.82)	0.0664
Adherence		−0.01 (−0.08, 0.05)	0.6587
**CESD**	**Mild to moderate depression versus Normal**	**0.31 (−0.01, 0.62)**	**0.0542**
**CESD**	**Major depression versus normal**	**1.21 (0.38, 2.04)**	**0.0045**
**Substance use**	**Yes versus no**	**−0.43 (−1.38, −0.26)**	**0.0044**
**Site**	**Brazil versus Thailand**	**1.56 (0.86, 2.25)**	**<0.00**
**Site**	**Zambia versus Thailand**	**−0.19 (−0.30, −0.08)**	**0.0010**
**Risk group**	**MSM versus HM**	**2.02 (1.13, 2.90)**	**<0.00**
Risk group	HW versus HM	−0.18 (−0.39, 0.02)	0.0747
**HIV transmission risk**		**Multivariable predictive model (backwards model selection, *p*)**	
Risk group within site			
Brazil	**MSM versus HM**	**2.85 (1.45, 4.25)**	**<0.0001**
	**MSM versus HW**	**3.37 (2.01, 4.74)**	**<0.0001**
	**HM versus HW**	**0.52 (0.28, 0.77)**	**<0.0001**
Thailand	MSM versus HM	0.10 (−0.27, 0.48)	0.5924
	MSM versus HW	−0.00 (−0.45, 0.45)	0.9979
	HM versus HW	−0.10 (−0.25, 0.04)	0.1572
Zambia	HM versus HW	−0.01 (−0.35, 0.33)	0.9515
Site within risk group			
MSM	**Thailand versus Brazil**	**−3.3 (−4.9, −1.78)**	**<0.0001**
HM	**Thailand versus Brazil**	**−0.59 (−0.82, −0.36)**	**<0.0001**
	Thailand versus Zambia	−0.09 (−0.42, 0.24)	0.5954
	**Brazil versus Zambia**	**0.50 (0.16, 0.84)**	**0.0043**
HW	Thailand versus Brazil	0.04 (−0.17, 0.25)	0.7211
	Thailand versus Zambia	0.00 (−0.21, 0.22)	0.9695
	Brazil versus Zambia	−0.03 (−0.23, 0.17)	0.7386
Energy/fatigue		−0.15 (−0.31, 0.02)	0.0787
Personal beliefs	Dislike of condoms	0.33 (−0.04, 0.70)	0.0794

MSM=men who have sex with men; HW=heterosexual women; HM=heterosexual men; CESD=Center for Epidemiological Studies Depression Scale. The bold values indicate variables that were statistically significant at *p*<0.05.

#### Multivariable model

When all predictors and interaction terms for site and risk group were entered into the model, following a backwards elimination selection, the only significant predictors were site×risk group interaction terms. Within study sites, in Brazil, MSM had more estimated HIV transmissions than heterosexual men and women. Additionally, heterosexual men had more estimated HIV transmissions than heterosexual women. In the other countries, there were no significant differences within each risk group. Within risk groups, for MSM, there were fewer estimated HIV transmissions in Thailand than Brazil. For heterosexual men, there were also fewer estimated HIV transmissions in Thailand compared with Brazil, and more estimated HIV transmissions in Brazil than Zambia. For heterosexual women, there were no site differences.

### STIs incidence and predictors

The STI incidence rate (over follow-up) of 22.4% occurred across the heterosexual women and MSM (Table 2). MSM in Thailand had the highest bacterial STI incidence (50.4%) followed by MSM in Brazil (34.1%), and heterosexual women in Thailand had the lowest (5.1%) (Table 2).

#### Bivariate model

In the bivariate model (Table 4), lower cognitive functioning, lower social support, higher scores on fear of consequences of HIV disclosure, higher alcohol use and higher depressive symptoms (severe depressive symptoms versus normal) were associated with higher bacterial STI incidence. There were also differences by site (Brazil and Zambia having fewer incident STIs than Thailand) and risk group (heterosexual women having fewer incident STIs than MSM).

**Table 4:** Predictors of STI incidence

STI incidence		Univariate model	
	
Characteristics	Level	OR (95% CI)	*p*
Time		1.00 (0.95, 1.05)	0.9993
General health		0.96 (0.88, 1.04)	0.3366
Physical functioning		1.08 (0.95, 1.22)	0.2293
Role functioning		0.98 (0.90, 1.06)	0.5532
Social functioning		0.93 (0.85, 1.02)	0.1291
**Cognitive functioning**		**0.88 (0.80, 0.96)**	**0.0034**
Pain		1.00 (0.92, 1.08)	0.9671
Mental health		1.08 (0.92, 1.28)	0.3407
Energy/fatigue		0.97 (0.89, 1.06)	0.5278
**Social support**		**0.96 (0.93, 0.99)**	**0.0112**
HIV disclosure behaviours		0.85 (0.54, 1.34)	0.4772
Fear of consequences of HIV disclosure		1.39 (0.95, 2.02)	0.0923
Community beliefs: power imbalance and condom negotiation		0.81 (0.63, 1.03)	0.0899
Community beliefs: relationship dynamics		0.88 (0.70, 1.09)	0.2447
Community beliefs: community negative beliefs about HIV		1.10 (0.90, 1.33)	0.3625
Community beliefs: sexual immorality		0.90 (0.71, 1.13)	0.3665
Personal beliefs: sexual practices		1.04 (0.85, 1.28)	0.7110
Personal beliefs: partner concerns		0.84 (0.67, 1.05)	0.1187
Personal beliefs: viral load beliefs		1.16 (0.94, 1.44)	0.1765
Personal beliefs: dislike of condoms		0.97 (0.80, 1.18)	0.7827
Adherence		0.94 (0.78, 1.13)	0.5236
**Alcohol score**		**1.06 (1.03, 1.10)**	**0.0005**
Substance use	No versus yes	0.66 (0.36, 1.18)	0.1585
CESD	Mild to moderate depressive symptoms versus normal	1.23 (0.76, 1.99)	0.4008
**CESD**	**Severe depressive symptoms versus normal**	**1.65 (1.02, 2.67)**	**0.0394**
**Site**	**Brazil versus Thailand**	**1.61 (1.01, 2.63)**	**0.0455**
	**Zambia versus Thailand**	**2.78 (1.33, 5.56)**	**0.0063**
**Risk group**	**MSM versus HW**	**4.76 (2.86, 7.69)**	**<0.00**
**STI incidence**		**Multivariate predictive model (backwards mode; selection, *p*)**	
Alcohol score		1.04 (1.01, 1.08)	0.0231
Risk group within site			
Brazil	MSM versus HW	1.93 (0.92, 4.04)	0.0804
**Thailand**	**MSM versus HW**	**12.3 (4.44, 33.98)**	**<0.0001**
Site within risk group			
**MSM**	**Thailand versus Brazil**	**2.63 (1.52, 4.57)**	**0.0006**
HW	Thailand versus Brazil	0.41 (0.13, 1.27)	0.1234
	Thailand versus Zambia	0.41 (0.13, 1.29)	0.1274
	Brazil versus Zambia	0.99 (0.40, 2.44)	0.9873

MSM=men who have sex with men; HW=heterosexual women; HM=heterosexual men; STI=sexually transmitted infection; CESD=Center for Epidemiological Studies Depression Scale. The bold values indicate variables that were statistically significant at *p*<0.05.

#### Multivariable model

In the multivariable model (Table 4), which included interaction terms for site and risk group, higher alcohol use remained significant associated with higher STI risk. Within site, in Thailand risk of STI was higher in MSM compared to heterosexual women (in the same direction for Brazil). Within the risk group, for MSM, Thailand had more incident STIs than Brazil, and there were no significant differences between sites for heterosexual women.

## Discussion

In this study, despite sampling individuals considered to be at the far right of the HIV care continuum because they are established and in HIV care, there were substantial numbers of estimated HIV transmissions. We selected for individuals who had admitted to some level of HIV transmission risk, and there were, overall, 2.20 estimated transmissions over the 12 months of study participation post-baseline for every 100 persons. Accordingly, secondary prevention interventions may impact HIV incidence when situated in the HIV care setting.

The groups and sites with higher numbers of estimated HIV transmissions (>3) were Brazilian MSM, Thai MSM, Brazilian women and Brazilian heterosexual men. MSM had the highest numbers of HIV transmissions and potentially are at high need for secondary prevention interventions, and of the countries investigated, Brazil appears to also be a location to prioritize. As there were not much findings with the predicted psychosocial variables, additional research is needed, potentially separately by risk group and/or site, to determine the factors contributing to these risk group×site interactions. In most of the HIV-negative samples, there are emerging data to suggest that HIV risk occurs in the context of additive but potentially different psychosocial problems, or syndemics, among women [18] and MSM [19,20], and it is possible that these three groups may experience different types of syndemic problems that could contribute to their risk behaviour among HIV-positive individuals.

The available STI data followed a similar but not identical pattern of results for region and risk group. Site differences were mainly among MSM, who had higher infections in Thailand versus Brazil. Risk-group differences were mainly among MSM and heterosexual women in Thailand. Alcohol use remained a significant predictor of STI incidence in the full model.

Various psychosocial predictors were significant in bivariate but not in multivariable models after accounting for risk group and site. Further research should tease out the degree to which these variables interact with risk across different settings and populations. Though depression, a potentially modifiable variable in the context of HIV care, did not emerge in the multivariable models, its significance in both models (estimated HIV transmissions and STI incidence), for example, speaks further to its importance in being addressed in TasP interventions.

For ethical reasons, participants were linked to existing standard of care and prevention counselling services at each site, and estimated HIV transmissions decreased during study participation. This is consistent with data from other HIV prevention studies showing declines in risk behaviours following enrolment due to either regression to the mean [21] or study participation [22–26]. Repeated inquiry via these assessments could also result in additional self-monitoring, leading, in turn, to a decrease in risk behaviour over time. Despite these factors, the number of estimated transmissions incident of STIs for the sample is high for the MSM and women where STIs were assessed longitudinally and likely low in heterosexual men where the baseline prevalence did not suggest high rates for longitudinal exploration. Accordingly, improving upon current standard of care and prevention counselling can augment TasP efforts.

We have several limitations to our study. Firstly, the estimated number of HIV transmissions is a composite variable relying on self-reported sexual behaviours as one component of its estimate. Although we used ACASI to minimize the potential for reporting bias, the actual numbers must therefore be interpreted with caution. However, due to perceived expectations for HIV-positive individuals, receiving counselling may result in some under-reporting, particularly in the visits after baseline. Additionally, it is possible that over- or under-reporting can occur as a function of the interview setting, and for the interviewer-administered components, who the interviewer is. Differences in willingness to report HIV transmission risk behaviours may explain some site differences as well. Secondly, due to low prevalence, we could not examine incident STIs among the heterosexual men. Thirdly, the Zambia site was not able to identify MSM at their clinic for potential participation. Finally, we had to use being on ART as a proxy for viral suppression in the estimation of potential transmissions.

## Conclusions

In the new era of ART for secondary HIV prevention, estimating HIV transmissions and monitoring incident STI infections is a more meaningful strategy for evaluating TasP than only focusing on self-reported condomless sex acts. Using estimated transmissions and incident STI as the basis for evaluating combined behavioural and biomedical TasP, interventions may be particularly useful when evaluating incremental cost-effectiveness of different types of TasP approaches, and to guide policy decisions regarding how and where to optimally implement TasP globally. The outcome of estimated transmissions may be a practical variable for examining HIV transmission risk in those living with HIV, likely, however, limited to phase 2 trials. Additionally, modelling potential transmissions may be important for other types of biomedical and behavioural HIV prevention programmes.

As enrolled in this study, identifiable patients are those who are sexually active and willing to self-report some level of risk, despite exposure to the standard of care and HIV risk reduction counselling. Our estimates suggest that augmenting secondary prevention programmes beyond messaging and providing education about HIV transmission by offering additional services such as evidence-based counselling for achieving viral suppression or reducing condomless sex, or support to patients who have co-occurring problems such as alcohol use or depression, and are sexually active and may be willing to admit to difficulties with HIV transmission risk behaviours may still be a means to decrease HIV incidence even in the era of ART for prevention.

## Supplementary Material

Frequency and predictors of estimated HIV transmissions and bacterial STI acquisition among HIV-positive patients in HIV care across three continents

Frequency and predictors of estimated HIV transmissions and bacterial STI acquisition among HIV-positive patients in HIV care across three continents
