# Supplementary material for: Frequency and predictors of estimated HIV transmissions and bacterial STI acquisition among HIV-positive patients in HIV care across three continents
Source: J Int AIDS Soc. 2016 Sep 28;19(1):21096. doi: 10.7448/IAS.19.1.21096 (PMC5043092; doi:10.7448/IAS.19.1.21096)
Supplement: Frequency and predictors of estimated HIV transmissions and bacterial STI acquisition among HIV-positive patients in HIV care across three continents [file JIAS-19-21096-s001.pdf]

Supplemental Tables.

1. **Principal Components Analysis: Four-factor Varimax rotated solutions**

**Perceived Community Beliefs**	**Factor 1**: Power Imbalance and Condom Negotiation	**Factor 2:** Sexual Expectations and Beliefs	**Factor 3:** Negative Community Beliefs about HIV	**Factor 4:** Sexual Immorality
9. Men fear that their partner(s) will leave them if they (the man) ask to use a condom.	**62**	4	-14	5
12. It is harder for younger people to ask their partners to use a condom if their partner is older.	**61**	13	1	-6
11. Women are obligated to be submissive to their husbands, and therefore cannot ask their husbands to use condoms.	**58**	4	5	-1
13. It is harder for people from a lower social status to ask their partners to use a condom if their partner is from a higher social status.	**56**	26	9	1
14. It is harder for people from ethnic or racial minority communities to ask their partners to use a condom if their partner is not a minority.	**54**	-12	17	-1
10. Women fear that their partner(s) will leave them if they (the woman) ask to use a condom.	**51**	**46**	-3	11
8. There is an obligation to have children and therefore engage in unprotected sex.	**49**	13	-21	20
15. People believe that only prostitutes use condoms.	**44**	-23	17	-5
16. The livelihood of individuals who engage in sex work is dependent on having unprotected sex.	**43**	-11	15	-9
7. There is an expectation to have unprotected sex once married.	15	**68**	9	11
5. It is difficult to introduce condoms in a relationship when the couple has already been having unprotected sex.	8	**63**	8	-6
21. Sex workers will lose income if they disclose that they are HIV positive.	-2	**61**	10	15
1. People believe it is prestigious for men to have multiple sex partners.	-2	**57**	7	-19
4. People believe that it is difficult for young people to have only one sexual partner.	8	**44**	9	-1
17. Condoms are not used often because they are too expensive.	26	**-49**	9	-25
20. People believe that women with HIV have engaged in sex with many partners.	6	17	**81**	-5

19. People believe that women with HIV have engaged in prostitution.	12	9	**81**	-2
18. It is believed that people with HIV have contracted the disease because they have engaged in immoral behavior.	5	14	**71**	11
2. People believe that men who have sex with multiple partners are immoral.	28	-27	4	**70**
3. People believe that women who have sex with multiple partners are immoral.	3	35	6	**64**
22. People believe that women cannot transmit HIV to others.	11	-5	-1	**-56**
6. Asking to use a condom suggests that you distrust your partner.	39	33	9	11
23. People who feel better after taking ART are more likely to have unsafe sex.	37	1	10	-31

**Personal Beliefs**	**Factor 1:** Sexual Practices	**Factor 2:** Partner Concerns	**Factor 3:** Viral load Beliefs	**Factor 4:** Dislike of Condoms
29. I only have sex with other HIV-infected people.	**69**	0	4	-11
39. I find it difficult to find a place to get condoms.	**68**	3	9	11
31. I only engage in unprotected sex in order to have a baby.	**62**	16	4	-12
30. I believe that condoms will protect me from STIs.	**-61**	-8	-17	5
38. I am embarrassed to get condoms at the store.	**60**	22	7	22
27. If I have unprotected sex with another HIV-infected person, I can acquire their type of the HIV virus.	**-54**	12	-26	2
32. I will lose status in my community if I am not a father or mother.	**50**	31	-12	10
26. Since HIV is treatable, I do not believe that it is important for me to use condoms.	**50**	1	**50**	14
28. If I am the receptive partner during sex (in other words, my partner is putting his penis inside of my mouth, anus, or vagina) I cannot transmit HIV to my partner.	**50**	-7	46	1
34. My partner(s) will think I am unfaithful if I ask to use a condom.	24	**85**	6	0
35. My partner(s) will think that I believe they are unfaithful if I ask to use a condom.	18	**84**	2	4
33. My partner(s) pressures me to have unprotected sex.	-9	**52**	8	8
25. I am more likely to engage in unsafe sex if I know that the amount of the HIV virus in my blood is undetectable.	9	14	**80**	13
24. I can't transmit HIV if the amount of the HIV virus in my blood is undetectable.	9	7	**84**	5

37. I feel that condoms make sex less pleasurable.	-13	-5	13	**83**
36. I believe that using condoms decreases intimacy between partners.	14	20	6	**81**

Note: Values are multiplied by 100 and rounded to the nearest integer.
